# Association between increased serum TNF-α levels and immediate memory impairment in patients with major depressive disorder: pilot study

**DOI:** 10.1192/bjo.2025.10908

**Published:** 2025-12-15

**Authors:** Gang Ye, Wen Long Hou, Jia Li, Li Juan Man, Zhen Hua Zhu, Xu Yuan Yin, Xin Yu, Huiping Zhang, Li Hui

**Affiliations:** Research Centre of Biological Psychiatry, Suzhou Guangji Hospital, Suzhou Medical College of Soochow University, Suzhou, PR China; Peking University Institute of Mental Health (the Sixth Hospital), Beijing, China; Departments of Psychiatry and Medicine (Biomedical Genetics), Boston University Chobanian & Avedisian School of Medicine, Boston, Massachusetts, USA

**Keywords:** TNF-α, major depressive disorder, cognitive function, immediate memory, association

## Abstract

**Background:**

Cognitive impairment in major depressive disorder (MDD) may be driven by neuro-inflammatory processes involving pro-inflammatory cytokines.

**Aims:**

This study aimed to examine the relationship between serum tumour necrosis factor-alpha (TNF-α) levels and cognitive performance across different domains in individuals with MDD.

**Method:**

Sixty patients with MDD and 60 healthy controls were recruited. Cognitive function was assessed using the Repeatable Battery for the Assessment of Neuropsychological Status (RBANS), and serum TNF-α levels were measured via flow cytometry.

**Results:**

After adjusting for covariates, RBANS total and subscale scores were significantly lower in MDD patients compared with controls (*P* < 0.001), while log_10_-transformed TNF-α levels were significantly higher in the MDD group (*P* = 0.006). In MDD patients, log_10_TNF-α levels were inversely correlated with immediate memory scores after adjusting for confounding factors (*r* = −0.35, *P* = 0.009); however, this relationship was not observed in healthy controls (*r* = −0.02, *P* = 0.90). Stepwise multivariate regression analysis further confirmed the negative association of log_10_TNF-α with immediate memory scores in MDD patients (*β* = −14.58, *t* = −4.14, *P* < 0.001), but not in healthy controls (*β* = −0.02, *t* = −0.14, *P* = 0.89).

**Conclusions:**

These findings suggest that elevated serum TNF-α may contribute to the pathophysiology of MDD and is specifically associated with deficits in immediate memory.

Major depressive disorder (MDD) is a highly prevalent and disabling psychiatric illness associated with significant morbidity and mortality,^1^ making it a major global public health concern. The lifetime prevalence of MDD ranges from 4.4 to 20% worldwide.^2^ In China it is the most common mood disorder, with a reported lifetime prevalence of 3.4% and a 12-month prevalence of 2.1%.^3^ While mood disturbances are central to MDD, cognitive impairment is also a well-established and clinically relevant feature.^4^ Affected cognitive domains include attention, memory, executive function, processing speed and psychomotor performance, and these deficits often persist beyond acute episodes into periods of remission.^5^ Cognitive impairment is not only common, but also independently contributes to functional disability in individuals with MDD. However, the underlying mechanisms of these cognitive deficits remain poorly understood.

One proposed mechanism involves neuro-inflammation mediated by cytokines such as tumour necrosis factor-alpha (TNF-α), a pro-inflammatory cytokine produced by macrophages, mast cells and natural killer cells.^6^ TNF-α is thought to contribute to the pathophysiology of MDD by promoting monoamine reuptake, activating the hypothalamic–pituitary–adrenocortical (HPA) axis and reducing serotonin (5-HT) synthesis through increased activity of indolamine-2,3-dioxygenase (IDO).^7^ Two recent meta-analyses demonstrated that TNF-α levels were significantly higher in patients with MDD in comparison with healthy controls.^8,9^ Beyond its role in inflammation and mood regulation, TNF-α also plays a critical role in central nervous system development and function, influencing neuronal plasticity, cognition and behaviour.^10^ Dysregulation of TNF-α signalling may impair hippocampal development and contribute to cognitive deficits.^11^ Human and animal studies have demonstrated inverse relationships between TNF-α levels and cognitive performance.^12,13^ For example, elevated plasma TNF-α concentration was negatively associated with memory performance in healthy adults,^14^ and higher peripheral TNF-α levels were associated with reduced processing speed, attention and executive functioning.^15^

Despite these findings, no studies to date have specifically investigated the relationship between peripheral blood TNF-α levels and distinct cognitive domains in individuals with MDD. Although there is currently no ‘gold standard’ for assessing cognitive impairment in MDD,^16^ the Repetitive Battery for the Assessment of Neuropsychological Status (RBANS) is a widely used tool that evaluates multiple cognitive domains.^17^ The present study aimed to examine whether serum TNF-α levels were associated with the impairment of specific cognitive domains among patients with MDD, as measured by RBANS.

## Method

### Ethics statement

All authors affirm that the procedures contributing to this study comply with the ethical standards of the relevant national and institutional committees on human experimentation, and with the principles of the Declaration of Helsinki (1975) as revised in 2013. The study protocol was approved by the Clinical Research Ethics Committee of Suzhou Guangji Hospital, affiliated with Suzhou Medical College of Soochow University (Approval no SGLL2020-005). This study was conducted between August 2020 and April 2024. Prior to participation, all individuals received a full explanation of the study protocol and procedures by a psychiatrist or trained research coordinator, and written informed consent was obtained from each participant.

### Participants

A total of 60 patients with MDD (28 males, 32 females) were recruited from Suzhou Guangji Hospital, affiliated with Suzhou Medical College of Soochow University. The inclusion criteria for the MDD group were as follows: (a) age between 18 and 70 years; (b) Han Chinese ethnicity; (c) diagnosis of MDD based on DSM-IV; (d) a minimum of 6 years of formal education; and (e) the ability to complete cognitive assessment.

Sixty healthy control participants (29 males, 31 females) were recruited from the local Suzhou community. Inclusion criteria for the control group were: (a) age between 18 and 70 years; (b) Han Chinese ethnicity; (c) a minimum of 6 years of formal education; and (d) the ability to complete cognitive assessment.

All participants were in good physical health at the time of the study. Exclusion criteria for both groups included a history of inflammatory conditions or use of medications with known immunomodulatory effects. Additional exclusions were diagnosis of dementia, neurodegenerative and neurological disorders, other psychiatric illnesses, drug or alcohol abuse, active infections, malignancies and pregnancy.

### Clinical measures

Demographic and clinical data were collected from all participants using a structured questionnaire. Cognitive functioning was assessed with RBANS, a brief and widely used neurocognitive battery designed for clinical settings. RBANS evaluates five cognitive domains: immediate memory, visuospatial/constructional abilities, language, attention and delayed memory.^18^ It comprises 12 subtests that yield 5 index scores and a total score. RBANS has demonstrated sensitivity to neuropsychological impairments across a range of neurological and psychiatric conditions, including depression.^19^ A validated Chinese version of RBANS has been developed, with established clinical utility and test–retest reliability in both health individuals and psychiatric populations.^17^

### TNF-α measurement

Blood samples (without anticoagulants) were collected from all MDD patients and healthy controls between 07.00 and 09.00 following an overnight fast. Serum was separated by centrifugation, aliquoted and stored at −80°C until analysis. Serum TNF-α levels were quantified using the BD™ Cytometric Bead Array Human Inflammatory Cytokines Kit (BD Biosciences, USA), in combination with a BD™ FACSCanto Flow Cytometer (BD Biosciences, USA), according to the manufacturer’s instructions. The kit has a reported sensitivity of 3.7 pg/mL for TNF-α, with intra- and inter-assay coefficients of variation of 9 and 8%, respectively. A standard curve was generated in triplicate using the standards provided for each assay batch. All assays were performed by the same technician, who was blinded to sample group assignments.

### Statistical analysis

Data were analysed using the Statistical Package for the Social Sciences (SPSS), version 24 for Windows. Demographic and clinical variables were compared between the MDD and healthy control groups using analysis of variance for continuous variables and chi-square tests for categorical variables. Because serum TNF-α levels were not normally distributed, they were log-transformed prior to analysis. Group differences in RBANS scores and log_10_-transformed TNF-α levels were assessed using analysis of covariance, adjusting for potential confounding factors. Pearson’s product–moment correlation coefficients were calculated to assess the associations between log_10_TNF-α levels and RBANS scores within each group. To further examine the predictive value of sociodemographic variables and log_10_TNF-α levels on cognitive performance, stepwise multivariate regression analyses were conducted separately for the MDD and control groups. All statistical tests were two-tailed, and a *P*-value of less than 0.05 was considered statistically significant.

## Results

### Demographic and clinical characteristics

The demographic and clinical characteristics are summarised in Table 1. Significant differences were observed between MDD patients and healthy controls in regard to age (*F* = 5.92, *P* = 0.02) and education level (*F* = 47.22, *P* < 0.001). However, there were no significant differences in gender, smoking status, alcohol consumption or body mass index (BMI; all *P* > 0.05). Among MDD patients, the mean ± standard deviation for age of illness onset, duration of illness and Hamilton Depression Scale (HAMD) scores was 35.46 ± 13.07, 5.83 ± 6.73 and 21.42 ± 5.76 years, respectively. Of the 60 patients, 33 were not taking antidepressants while 27 were on prescribed medications, including selective serotonin reuptake inhibitors (*n* = 12), serotonin/norepinephrine reuptake inhibitors (*n* = 6), other single antidepressants (*n* = 3) and combined antidepressants (*n* = 6).

**Table 1:** Demographic and clinical variables in MDD patients and healthy controls

Variable	MDD patients (*n* = 60)	Healthy controls (*n* = 60)	*F* or *χ* ^2^	*P*-value
Gender (male/female)	28/32	29/31	0.03	0.86
Age (years)	41.48 ± 13.40	35.72 ± 12.54	5.92	0.02
Education (years)	8.85 ± 3.16	12.78 ± 3.11	47.22	<0.001
Smoking (smoker/non-smoker)	14/46	12/48	0.20	0.66
Drinking (drinker/non-drinker)	11/49	6/54	1.71	0.19
BMI (kg/m^2^)	22.35 ± 3.59	22.76 ± 3.13	0.43	0.51
Age of onset (years)	35.46 ± 13.07			
Duration of illness (years)	5.83 ± 6.73			
HAMD score	21.42 ± 5.76			
Antidepressant type				
SSRIs	12			
SNRIs	6			
Drug combination	6			
Other drugs	3			
No drugs	33			

MDD, major depressive disorder; BMI, body mass index; HAMD, Hamilton Depression Scale; SSRIs, selective serotonin reuptake inhibitors; SNRIs, serotonin/norepinephrine reuptake inhibitors; drug combination, two or more antidepressants; other drugs, those such as tricyclic antidepressants and noradrenergic and specific serotonergic antidepressants.

### Comparison of RBANS scores and serum TNF-α levels

**Fig. 1:**
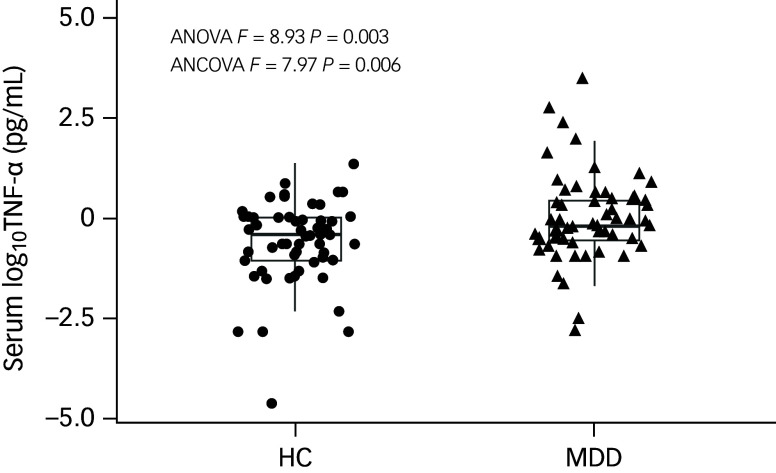
Comparison of serum log_10_TNF-α levels between major depressive disorder patients (MDD) and healthy controls (HC). Serum log_10_TNF-α levels were significantly higher in patients with MDD than healthy controls after adjusting for covariates (−0.01 ± 0.47 *v*. −0.26 ± 0.44, *F* = 7.97, *P* = 0.006). TNF-α, tumour necrosis factor-alpha; ANOVA, analysis of variance; ANCOVA, analysis of covariance.

The RBANS index and total scores of both groups are presented in Table 2. All RBANS scores were significantly lower in MDD patients compared with healthy controls (*P* <0.001 for all comparisons). These differences remained significant after adjusting for gender, age, education, smoking, drinking and BMI (*P* < 0.001 for all). Education significantly influenced all RBANS scores (*P* < 0.05) except for delayed memory scores (*F* = 2.64, *P* = 0.11). Additionally, age (*F* = 7.19, *P* = 0.008) and drinking status (*F* = 7.08, *P* = 0.009) had significant effects on attention scores between the two groups. Serum TNF-α levels were not normally distributed and were therefore log-transformed. As shown in Fig. 1, log-transformed TNF-α (log_10_TNF-α) levels were significantly higher in MDD patients compared with healthy controls (−0.01 ± 0.47 *v*. −0.26 ± 0.44, *F* = 8.93, *P* = 0.003). This difference remained significant after adjusting for covariates (*F* = 7.97, *P* = 0.006).

**Table 2:** Comparisons of RBANS index and total scores between MDD patients and healthy controls

Index	MDD patients (*n* = 60)	Healthy controls (*n* = 60)	*F*	*P*-value	Adjusted *F* ^a^	Adjusted *P* ^*a*^-value
Immediate memory	59.55 ± 11.76	92.90 ± 17.59	149.05	<0.001	24.26	<0.001
Visuospatial/constructional	68.90 ± 11.67	82.13 ± 12.28	36.62	<0.001	7.31	<0.001
Language	73.15 ± 13.51	94.82 ± 14.85	69.87	<0.001	14.42	<0.001
Attention	84.93 ± 19.00	111.80 ± 15.21	73.11	<0.001	21.67	<0.001
Delayed memory	70.87 ± 13.56	94.03 ± 7.95	130.34	<0.001	19.72	<0.001
RBANS total score	357.40 ± 45.62	475.68 ± 41.87	218.94	<0.001	47.77	<0.001

RBANS, Repetitive Battery for the Assessment of Neuropsychological Status; MDD, major depressive disorder.

a. Adjusted *F* and *P*-values indicate the respective values after controlling for gender, age, education, smoking, drinking and body mass index.

### Associations between RBANS scores and serum TNF-α levels

**Fig. 2:**
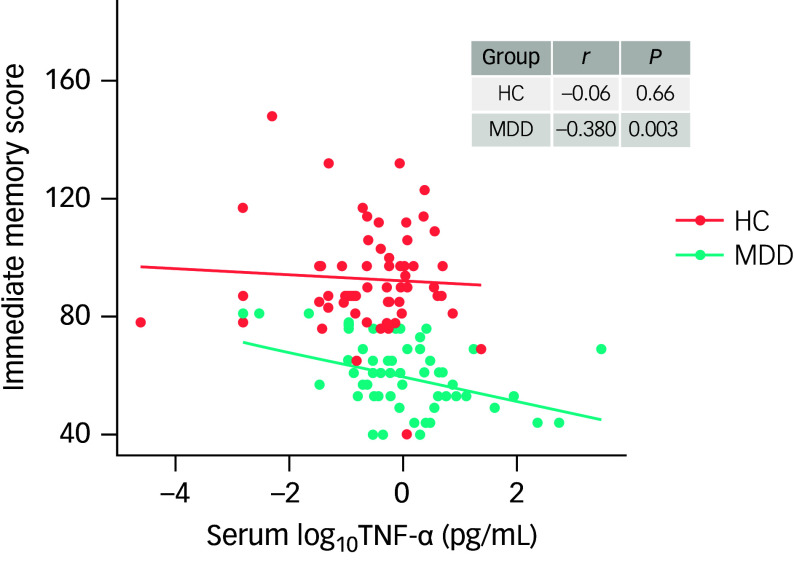
A significantly negative correlation between log_10_TNF-α levels and immediate memory score was found for major depressive order patients (MDD) (*r* = −0.38, *P* = 0.003), but not for healthy controls (HC) (*r* = −0.06, *P* = 0.66). TNF-α, tumour necrosis factor-alpha.

As shown in Fig. 2, Pearson correlation analysis revealed a significant negative correlation between log_10_ TNF-α levels and immediate memory scores in MDD patients (*r* = −0.38, *P* = 0.003), but not in healthy controls (*r* = −0.06, *P* = 0.66). After adjusting for gender, age, education, smoking, drinking, BMI, age of onset, illness duration, HAMD scores and antidepressant use, this association remained significant in MDD patients (*r* = −0.35, *P* = 0.009) but not in healthy controls (*r* = −0.02, *P* = 0.90). No significant associations were observed between log_10_TNF-α levels and other cognitive domain scores in either group (all *P* > 0.05).

Furthermore, stepwise multivariate regression analysis indicated that log_10_TNF-α levels (*β* = −14.58, s.e. = 3.52, *t* = −4.14, *P* < 0.001, 95% CI: −21.75 to −3.83) and antidepressant use (*β* = −12.79, s.e. = 4.37, *t* = −2.93, *P* = 0.007, 95% CI: −21.80 to −7.36) were significantly associated with immediate memory scores in MDD patients (Table 3). In contrast, no significant association between log_10_TNF-α levels and immediate memory scores was found in healthy controls (*β* = −0.02, *t* = −0.14, *P* = 0.89). Additionally, there were no significant associations between TNF-α levels and other RBANS scores in either group (all *P* > 0.05).

**Table 3:** Stepwise multivariate regression model of social-demographic and log_10_TNF-α determinants of immediate memory score in MDD patients

Variables	*β*	*t*	*P*
Gender	0.28	1.98	0.06
Age	−0.17	−1.15	0.26
Education	−0.02	−0.10	0.92
Smoking	−0.25	−1.77	0.09
Drinking	0.13	0.85	0.40
BMI	−0.04	−0.27	0.79
Age of onset	−0.19	−1.31	0.20
Duration of illness	0.03	0.16	0.87
HAMD score	−0.16	−1.01	0.32
Drug or not	−12.79	−2.93	0.007
log_10_TNF-α	−14.58	−4.14	<0.001

TNF-α, tumour necrosis factor-alpha; MDD, major depressive disorder; BMI, body mass index; HAMD, Hamilton Depression Scale.

## Discussion

To our knowledge, this is the first study to examine the association between serum TNF-α levels and cognitive function, assessed by RBANS, in Han Chinese patients with MDD. Our findings suggest that patients with MDD exhibit significantly poorer performance across all cognitive domains compared with healthy controls, and that elevated serum TNF-α levels are negatively correlated with immediate memory scores in MDD. Furthermore, serum TNF-α levels were significantly higher in MDD patients than in healthy controls.

Cognitive dysfunction is increasingly recognised as a core feature of MDD. In this study, Han Chinese patients with MDD demonstrated impairments in immediate memory, visuospatial–constructional, language, attention and delayed memory. These findings are consistent with prior studies showing that MDD affects multiple cognitive domains across all age groups,^20–22^ and persists during the acute and/or remission phases of the disorder.^23,24^ Notably, cognitive impairments often continue even after affective symptoms have been resolved.^25^ This underscores the importance of targeting cognitive function early in the course of MDD. Early identification and intervention may reduce cognitive decline and decrease the risk of chronic relapse and recurrence.^26^

TNF-α, a pro-inflammatory cytokine released during neuronal activity, plays a crucial role in modulating synaptic strength.^27^ Our results showed a significant negative correlation between serum TNF-α levels and immediate memory performance in MDD patients. This aligns with previous research suggesting that dysregulated TNF-α signalling may contribute to impaired hippocampal development and cognitive dysfunctions.^28^ TNF-α has been found to interact with brain-derived neurotrophic factor (BDNF) during memory formation,^29–31^ and basal TNF-α levels are believed to be essential for early cognitive development by influencing the expression of neurotrophins such as BDNF and nerve growth factors.^32^

Additionally, studies have observed interactive effects between TNF-α and BDNF gene polymorphisms on memory retention, indicating that elevated TNF-α may have neurotoxic effects.^33^ In a rodent model of multiple sclerosis, TNF-α released by astrocytes altered excitatory synapses in the hippocampus, potentially impairing synaptic plasticity and memory.^34^ Increased TNF-α has also been reported to inhibit long-term potentiation (LTP), reduce synaptic plasticity and decrease hippocampal volume via activation of the neurodegenerative TNFRSF1A pathway, thereby affecting memory formation.^35–37^ However, some animal studies have reported that TNF-α is involved in neither LTP induction nor maintenance,^38,39^ and TNF-α deficiency has been associated with declines in memory-related function.^40,41^ These conflicting findings may be explained by the region-specific expression and function of TNF-α in the brain.^42^

Neuro-inflammation is one of several interrelated neurobiological mechanisms implicated in the pathophysiology of MDD. Numerous clinical studies have reported abnormal inflammation profiles in both the brain and peripheral tissues of patients with MDD.^43^ Consistent with this, our data showed significantly elevated serum TNF-α levels in MDD patients, in line with a recent meta-analysis confirming higher TNF-α levels in patients with MDD in comparison with healthy subjects.^44^ At the molecular level, a genome-wide association study identified TNF-α polymorphisms associated with MDD,^45^ suggesting a potential role of TNF-α in the pathophysiology of the disorder.

TNF-α may contribute to the pathogenesis of depression by enhancing monoamine reuptake, activating the HPA axis and reducing serotonin (5-HT) synthesis via the increased activity of IDO, a rate-limiting enzyme in the kynurenine pathway.^7^ The comorbidity between MDD and cognitive deficits may be mediated by shared inflammatory mechanisms, including cytokine-induced dysregulation of the kynurenine pathway.^46^ Chronic TNF-α-mediated upregulation of IDO can cause a lasting shift towards kynurenine production, resulting in reduced 5-HT levels and elevated kynurenines in the brain.^7,46^ Increased levels of kynurenic acid in the prefrontal cortex, reported in schizophrenia, have been linked with disturbances in glutamate, dopamine and acetylcholine signalling, all of which are crucial for cognitive function.^47^ These findings further support the hypothesis that elevated TNF-α may mediate the link between depression and cognitive impairment.

### Limitations

This study has several limitations. First, the relatively small sample size and the cultural homogeneity of subjects may have limited the generalisability of the findings and increased the risk of statistical bias. Second, the cross-sectional design precludes any conclusions about causality between elevated TNF-α levels and immediate memory impairment in patients with MDD. Future research using larger, longitudinal cohorts is needed to clarify the directionality and causative mechanisms. Third, our sample included a mix of first-episode and recurrent MDD cases, making it difficult to control for potential confounding factors such as the number of episodes and medication history. Fourth, RBANS does not assess certain cognitive domains, such as executive functioning, which are also shown to be affected in depression. Finally, we measured TNF-α levels only in peripheral blood, which may not accurately reflect central nervous system activity or neuro-inflammation within the brain.

### Implications

Our findings suggest that serum TNF-α levels are elevated in MDD patients compared with healthy controls and are negatively correlated with immediate memory performance, indicating a potential role of TNF-α in the pathogenesis and cognitive impairment associated with MDD. Additionally, patients with MDD demonstrated poorer cognitive performance across multiple domains. While these results are promising, they should be interpreted with caution due to the relatively small sample size and the cross-sectional design of the study. Future research with larger, independent cohorts and longitudinal follow-up is warranted to validate these findings and further explore the mechanistic role of TNF-α in depression-related cognitive dysfunction.

## Data Availability

Raw data from this study are available from the corresponding author, L.H., on reasonable request.
